# Associations between cognitive–personality traits and perceptual responses during low-volume high-intensity interval exercise in overweight-to-obese adults

**DOI:** 10.3389/fpsyg.2025.1744648

**Published:** 2026-01-12

**Authors:** Ruohan Zhang, Jingyuan Sun, Jinfa Gu

**Affiliations:** Exercise and Sports Science Programme, School of Health Sciences, Universiti Sains Malaysia, Kota Bharu, Kelantan, Malaysia

**Keywords:** affective response, cognitive–personality traits, low-volume high-intensity interval exercise, perceptual responses, psychological tolerance

## Abstract

**Background:**

Low-volume high-intensity interval exercise (Lv-HIIE) has been proposed as a time-efficient, effective training approach for adults with overweight or obesity. However, its perceptual tolerability may depend on cognitive–personality traits influencing emotional and exertional regulation. This study examined how these traits relate to perceptual responses during Lv-HIIE.

**Methods:**

Thirty-two physically inactive adults (BMI = 28.21 ± 2.93 kg·m^2^; 11 males, 21 females; age = 28.3 ± 4.9 years) completed a 10-week Lv-HIIE program of 30 supervised sessions. Perceptual measures of affective valence, arousal, perceived exertion, recovery, and enjoyment were collected in every session, and data from the 1st, 15th, and 30th sessions were analyzed as representative points. Goal orientation and personality hardiness were assessed before the intervention, and Pearson’s correlations examined associations between perceptual responses and cognitive–personality variables.

**Results:**

Across the intervention, affective valence showed strong negative correlations with heart rate and perceived exertion, and positive correlations with enjoyment (*r* = −0.82 to −0.66, *p* < 0.01). Goal orientation and hardiness were moderately to strongly associated with higher affective valence (*r* = 0.59–0.65) and greater enjoyment (*r* = 0.38–0.56), while correlating negatively with perceived exertion (*r* = −0.38 to −0.56). These associations became progressively stronger toward later sessions, indicating adaptive affective regulation and enhanced psychological tolerance with repeated Lv-HIIE exposure.

**Conclusion:**

Individuals with higher goal orientation and greater hardiness exhibited more positive affective and enjoyment responses together with lower perceived exertion. Overall, these findings suggest that cognitive–personality traits contribute to perceptual and affective regulation during high-intensity exercise.

## 1 Introduction

The global prevalence of overweight and obesity has increased dramatically, posing a serious challenge to public health systems. The World Obesity Report (2025) projects that the number of adults with obesity will rise from 524 million in 2010 to 1.13 billion by 2030, suggesting that nearly half of the world’s adult population will be affected by excess body weight (World Obesity Federation, 2025). In Malaysia, according to recent studies, approximately 31.3% of adults are overweight and 22.2% obese (National Health and Morbidity Survey, 2023; Malaysian Association for the Study of Obesity, 2022), while 43.7% are physically inactive (Malaysian Association for the Study of Obesity, 2022). The situation underscores the urgent need for effective and engaging exercise strategies for this population.

With the rise of lifestyle-related diseases, public health initiatives are increasingly focusing on developing exercise programs that are efficient, enjoyable, and sustainable (Batrakoulis et al., 2024). Although regular physical activity yields extensive physical and psychological benefits (Kylasov and Gavrov, 2020), global participation remains low, with 31% of adults and 80% of adolescents failing to meet WHO recommendations (World Health Organization, 2024). “Lack of time” remains a dominant barrier (Hoare et al., 2017; Heesch and Mâsse, 2004; Cavallini, 2022; Atakan et al., 2021). Moreover, traditional moderate-intensity continuous exercise (MICE) often leads to modest weight loss (Shaw et al., 2006; Wu et al., 2009), emphasizing the need for time-efficient and psychologically acceptable alternatives that maintain adherence and positive engagement.

High-intensity interval exercise (HIIE) is recognized as a time-efficient training strategy that improves cardiorespiratory fitness and reduces body fat within short periods (Andreato et al., 2019; Batacan et al., 2017; Wewege et al., 2017). However, its demanding nature often induces discomfort and negative affective experiences, particularly among individuals unaccustomed to vigorous exertion (Weston et al., 2014; Hardcastle et al., 2014; Niven et al., 2021). To enhance tolerability, low-volume HIIE (Lv-HIIE) was developed to reduce total duration and perceived strain while maintaining comparable health benefits (Sabag et al., 2022; Yin et al., 2024), offering a potentially sustainable alternative for adults living with excess body weight.

However, adherence to Lv-HIIE cannot be guaranteed because an individual’s perceptions and psychological experiences during the exercise play a crucial role in determining long-term participation. Adherence to such protocols depends on both physiological capacity and perceptual–psychological factors. Affective responses, defined as the pleasure or displeasure experienced during activity, are robust predictors of future participation (Ekkekakis et al., 2008; Ekkekakis et al., 2011). According to the Dual-Mode Theory (Ekkekakis et al., 2005), these responses arise from interactions between interoceptive cues and higher-order cognitive processes. Among adults with obesity, heightened discomfort and perceived exertion may undermine enjoyment and adherence, making it essential to understand the cognitive mechanisms that shape perceptual experiences during high-intensity exercise. Despite growing evidence linking affective responses to adherence, limited research has examined how cognitive–personality traits influence perceptual responses in this context.

Cognitive–personality traits represent stable motivational and interpretive styles that influence how individuals perceive, cope with, and respond to stressors (Lazarus and Folkman, 1987; Cloninger, 2004). Within exercise settings, such traits modulate the balance between challenge and threat appraisals, influencing affective and motivational outcomes (Ryan and Deci, 2000; Gaudreau and Blondin, 2002). Two key traits, goal orientation and hardiness, have been identified as major determinants of affective and motivational responses to demanding tasks. Goal orientation theory (Nicholls, 1984; Duda and Nicholls, 1992) makes a distinction between task and ego orientations. Generally, when performing exercise, task-oriented individuals report greater enjoyment, persistence, and positive affect, while ego-oriented individuals experience more anxiety and negative affect. Hardiness, characterized by commitment, control, and viewing challenges as growth opportunities (Kobasa, 1979), is associated with adaptive coping and emotional stability under stress. Together, these traits may influence how adults with excess body weight interpret and regulate high-intensity exercise experiences. Accordingly, this study examines the relationships between goal orientation, hardiness, and perceptual responses, including affective valence, perceived exertion, and enjoyment during Lv-HIIE, aiming to clarify the cognitive factors underpinning exercise motivation and adherence.

## 2 Methods

### 2.1 Participants

Thirty-two physically inactive adults (11 males, 21 females; age 23–33 years) were included in the final analysis. According to the WHO Asian body mass index (BMI) classification, 15.6% were overweight (BMI = 23–24.9 kg·m^2^) and 84.4% had obesity (BMI ≥ 25 kg·m^2^). All participants engaged in less than 150 min of moderate physical activity per week and were recruited from Universiti Sains Malaysia. Sample size estimation using G*Power (v3.1.9.2) indicated that 31 participants were required to detect a moderate effect size (*F* = 0.30; *α* = 0.05; power = 0.80), based on prior studies (Astorino et al., 2019; Martinez-Diaz et al., 2021). Inclusion criteria were adults aged 20–35 years with BMI 23–30 kg·m^2^, medically fit but inactive. Exclusion criteria included smoking, metabolic disorders, medication affecting exercise response, recent structured training, or contraindications to exercise (PAR-Q). The study was approved by the Human Research Ethics Committee, Universiti Sains Malaysia (JEPeM Code: USM/JEPeM/22080549), and all participants provided written informed consent.

### 2.2 Experimental overview

This interventional study employed a within-subjects design in which each participant completed a similar Lv-HIIE protocol across a 10-week training intervention. Prior to the intervention, participants attended a preliminary session to measure anthropometric variables and assess cardiorespiratory fitness. After a minimum of 48 h, they completed a 20 m shuttle run test (pre-HIIE) to establish baseline performance. Participants then engaged in a 10-week Lv-HIIE program consisting of three supervised sessions per week (total of 30 sessions), with at least 48 h between sessions. All sessions were conducted at the Sports Complex, Health Campus, Universiti Sains Malaysia, in an outdoor environment under consistent conditions. To minimize confounding factors related to environmental and biological variation, participants performed all exercise sessions at the same location and time of day (between 8:00 a.m. and 12:00 p.m.). Perceptual responses, including affective valence (Feeling Scale), felt arousal (FAS), rating of perceived exertion (RPE), and heart rate (HR), were measured during each session. Enjoyment (PACES) was recorded at the end of the 1st, 15th, and 30th sessions, while perceived recovery status (PRS) and heart rate recovery (HRR) were assessed after each session. Personality hardiness and goal orientation were evaluated before the training intervention. Participants received standardized verbal instructions on the use of each scale during the familiarization phase, and all measurements were administered by the same researcher to ensure consistency.

#### 2.2.1 Anthropometric and physical activity measures

Stature and body mass were measured to the nearest 0.1 cm and 0.1 kg, respectively, using a stadiometer and digital scale (Seca 220, Hamburg, Germany), with participants barefoot and wearing light clothing. Waist-to-hip ratio (WHR) was determined by dividing waist circumference (cm) by hip circumference (cm). BMI was calculated as body mass (kg) divided by stature squared (m^2^). Percentage body fat (%BF) was assessed using a foot-to-foot bioelectrical impedance analyzer (Tanita TBF-140, Japan), which has been validated for estimating %BF in overweight and obese adults (Li et al., 2013).

Participants’ habitual physical activity levels were assessed using the English version of the International Physical Activity Questionnaire (IPAQ-M; Shamsuddin et al., 2015). The questionnaire was selected because it had demonstrated good validity and reliability among Malaysian adults in the previous analysis (Chu and Moy, 2015). The IPAQ-M classifies physical activity into three categories: inactive (<600 MET·min/week), moderately active (600–3,000 MET·min/week), and health-enhancing physical activity (>3,000 MET·min/week) (Macfarlane et al., 2007).

#### 2.2.2 Cardiorespiratory fitness

Participants performed an incremental treadmill test (1% gradient) to determine maximal oxygen uptake (*V̇*O₂max) and ventilatory threshold (VT). The test started at 4 km/h, with 0.5 km/h increments every 30 s until exhaustion. Simultaneously, the gas exchange was measured continuously (Cortex Metalyzer III B, Germany), and HR was monitored (Polar Electro, Finland). *V̇*O₂max was defined as the highest 10-s average *V̇*O₂ with a respiratory exchange ratio ≥1.10 and HR within 5 beats of age-predicted HRmax. VT was identified via ventilatory equivalents for *V̇*O₂ and *V̇*CO₂. This treadmill protocol has been validated in prior studies for assessing *V̇*O₂max in adults with overweight and obesity (Balcı, 2012; Khammassi et al., 2018).

#### 2.2.3 HIIE protocols

Participants performed a 10-week Lv-HIIE intervention adapted from previous Lv-HIIE models for overweight adults (Sabag et al., 2022; Yin et al., 2024). Each session consisted of 6–10 repetitions of 1-min work intervals performed at 90% of maximal aerobic speed (MAS), settings determined from the pre-intervention 20 m shuttle run test. The number of repetitions followed a predefined, time-based progression applied uniformly to all participants. Specifically, participants completed six repetitions per session during weeks 1–2, seven repetitions during weeks 3–4, with the number of repetitions increasing by one every 2 weeks, reaching 10 repetitions per session during weeks 9–10. This progression was designed to provide a gradual increase in training volume, allowing participants to adapt to the physiological demands of Lv-HIIE while maintaining exercise tolerance and safety. This progression was implemented for all participants and was not individually adjusted based on fitness changes. Each work interval was interspersed with 75 s of active recovery at a self-paced walking speed. A 3-min warm-up and 2-min cool-down at self-paced running were completed before and after each session. Participants performed the Lv-HIIE protocol three times per week for 10 weeks (30 sessions in total). All training sessions were conducted under consistent outdoor conditions and supervised by the same investigator to ensure standardized exercise execution and participant safety.

### 2.3 Experimental measures

#### 2.3.1 HR response

HR was continuously monitored during each Lv-HIIE session using a telemetry system (Polar Electro, Kempele, Finland). Working HR was recorded throughout all work and recovery intervals to ensure that exercise intensity corresponded to approximately 90% of maximal aerobic speed. Meanwhile, recovery HR was measured during the 75-s active recovery periods and within the first minute post-exercise to evaluate cardiovascular recovery dynamics across sessions. All HR data were averaged over 10-s intervals and analyzed to monitor training intensity and adaptation throughout the 10-week intervention.

#### 2.3.2 Goal orientation in exercise

Individual goal orientation was measured at baseline using the Goal Orientation in Exercise Measure (GOEM; Petherick and Markland, 2008). The GOEM consists of 10 items assessing how individuals define success in exercise and includes task-oriented (e.g., “I exercise to the best of my ability”) and ego-oriented (e.g., “I know that I am more capable than other exercisers”) dimensions. Responses were rated on a five-point Likert scale ranging from 1 (strongly disagree) to 5 (strongly agree). In the present study, goal orientation was operationalized using an overall composite GOEM score rather than analyzing task- and ego-oriented dimensions separately. The overall GOEM score was calculated by summing all 10 items, yielding a possible score range of 10–50, with higher scores indicating stronger overall achievement goal orientation. This approach was adopted to provide a global index of goal orientation and to maintain adequate statistical power given the modest sample size. The scale demonstrated good internal consistency, with Cronbach’s *α* values of 0.78 for the task subscale and 0.88 for the ego subscale.

#### 2.3.3 Personality hardiness in exercise

Hardiness was assessed using the Connor-Davidson Resilience Scale (CD-RISC; Connor and Davidson, 2003), a 25-item self-report questionnaire measuring an individual’s ability to cope with stress and adversity. Each item was rated on a 5-point Likert scale ranging from 0 (“Not true at all”) to 4 (“True nearly all the time”), with higher total scores indicating greater resilience. An example of an item is “I am able to adapt when changes occur.” The CD-RISC has demonstrated strong psychometric properties in prior research and showed high internal consistency in the present study (Cronbach’s *α* = 0.91). This measure was administered at baseline before the Lv-HIIE intervention.

#### 2.3.4 Affective responses

Affective valence, reflecting the degree of pleasure or displeasure during exercise, was assessed using the Feeling Scale (FS; Hardy and Rejeski, 1989). Participants rated their current feelings on an 11-point bipolar scale ranging from +5 (“Very Good”) to −5 (“Very Bad”). Perceived activation was measured using the FAS Scale (FAS; Svebak and Murgatroyd, 1985), a single-item 6-point scale ranging from 1 (“Low Arousal”) to 6 (“High Arousal”). Both FS and FAS have demonstrated good convergent validity with the Affect Grid (*r* = 0.41–0.65; Russell et al., 1989; Van Landuyt et al., 2000) and were jointly used to assess affective responses within the circumplex model of affect (Russell et al., 1989). The FS and FAS were measured at three standardized time points: 5 min before exercise, 20 s before the end of the warm-up, and 20 s before the end of each work interval. For sessions 1, 15, and 30, the FS and FAS values used for correlation analyses were calculated as the mean of all measurements obtained during the work intervals within each session, providing a session-level affective response indicator.

#### 2.3.5 Rating of perceived exertion

Perceived exertion during exercise was assessed using the 10-point Category-Ratio Scale (CR-10; Borg, 1998), commonly known as the Rating of Perceived Exertion (RPE). The scale ranges from 0 (“No exertion at all”) to 10 (“Maximal exertion”), reflecting participants’ subjective effort levels. RPE was assessed at the same standardized time points as affective responses, specifically 20 s before the end of each work interval. When multiple perceptual measures were collected at the same time point, RPE was assessed first, followed by the FS and the FAS.

#### 2.3.6 Post-exercise enjoyment

Exercise enjoyment was assessed 10 min post-exercise using the Physical Activity Enjoyment Scale (PACES; Kendzierski and DeCarlo, 1991). The PACES includes 18 items rated on a 7-point bipolar scale (e.g., “It’s not very refreshing/It’s very refreshing”). Total scores range from 18 to 126, with higher scores indicating greater enjoyment. Internal consistency was high across all assessments (Cronbach’s *α* > 0.90). Additionally, PACES were administered after each session on the first, fifteenth, and final days of the Lv-HIIE intervention.

#### 2.3.7 Perceived recovery status

Perceived recovery was assessed using the Perceived Recovery Scale (PRS), a single-item 0–10 scale evaluating recovery and expected performance, where 0 indicates “very poorly recovered (poor performance)” and 10 indicates “fully recovered (optimal performance).” The PRS has been widely used as a simple subjective indicator of recovery status in exercise settings.

### 2.4 Statistical analysis

All statistical analyses were conducted using SPSS version 28.0 (IBM Corporation, Armonk, NY, USA). The Shapiro–Wilk test was used to verify the normality of distribution for all dependent variables. Descriptive characteristics (mean ± standard deviation) between pre-test and post-test measurements were analyzed using paired samples t-tests. Pearson’s product–moment correlation coefficients (*r*) were computed to examine: (a) the relationships among perceptual responses during Lv-HIIE, specifically affective valence, perceived exertion, perceived recovery, and enjoyment; and (b) the associations between these perceptual responses and cognitive traits, including goal orientation and personality hardiness. Correlation coefficients were interpreted as follows: 0.10–0.39 (weak), 0.40–0.69 (moderate), and 0.70–1.00 (strong) (Cohen, 1988). Statistical significance was set at *p* < 0.05.

All figures were generated using Origin software (OriginLab Corporation, Northampton, MA, USA) solely for data visualization and figure preparation; no statistical analyses were conducted in Origin.

## 3 Results

The participants’ descriptive characteristics are shown in Table 1. Thirty-two adults (11 males, 21 females; age = 28.3 ± 4.9 years) completed the study. Mean height and body weight were 162 ± 8.6 cm and 74.19 ± 11.22 kg, respectively, yielding a BMI of 28.21 ± 2.93 kg·m^−2^ (overweight-to-obese range). Significant reductions were observed in body-fat percentage (32.89 ± 5.39 to 31.78 ± 5.51%; *p* = 0.003) and waist-to-hip ratio (0.81 ± 0.08 to 0.78 ± 0.07; *p* = 0.013). Cardiorespiratory fitness improved, with *V̇*O₂max increasing from 38.15 ± 2.28 to 38.55 ± 2.44 mL·kg^−1^·min^−1^ (*p* = 0.002) and MAS from 9.55 ± 0.68 to 10.03 ± 0.76 km·h^−1^ (*p* < 0.001). Participants were generally low-active before the intervention (518 ± 334 MET-min·week^−1^). However, all of them successfully completed the 10-week Lv-HIIE program without adverse events.

**Table 1:** Descriptive characteristics of the participants (*N* = 32).

Variables	Mean ± SD			*p*-value	F/ES
	Pre-test	Mid-test	Post-test		
Age (years)	28.3 ± 4.9	–	–	–	–
Height (cm)	162 ± 8.6	–	–	–	–
Body weight (kg)	74.19 ± 11.22	73.92 ± 10.78	73.93 ± 11.25	0.63	0.45
BMI (kg/m^2^)	28.21 ± 2.93	28.08 ± 2.8	28.12 ± 2.85	0.61	0.45
%BF (%)	32.89 ± 5.39	32.04 ± 5.31	31.78 ± 5.51*	0.003	6.98
WHR	0.81 ± 0.08	0.79 ± 0.09	0.78 ± 0.073*	0.013	5.22
*V̇*O₂max	38.15 ± 2.28	–	38.55 ± 2.44^	0.002	0.62
MAS	9.55 ± 0.68	–	10.03 ± 0.76^	<0.001	1.8
HR_max_	184 ± 10.25	–	180.63 ± 9.31^	<0.03	0.43
IPAQ (MET-min/week)	518 ± 334	–	–	–	–

Values are reported as mean ± standard deviation. BMI, Body mass index; BF, Body fat; WHR, Waist hip ratio; *V̇*O₂max, Maximal oxygen uptake; MAS, maximum aerobic speed; HRmax, maximum heart rate; IPAQ, International Physical Activity Questionnaire; *Significant differences between all-time points (*p* < 0.05); ^Significant difference between pre- and post-intervention (*p* < 0.05).

### 3.1 Correlations between perceptual responses during Lv-HIIE

For all correlation analyses, Pearson’s correlation coefficient (*r*) was used to quantify the magnitude of associations (i.e., effect size), with effect strength interpreted using conventional thresholds (|*r*| ≈ 0.10 small, 0.30 moderate, and ≥0.50 large).

#### 3.1.1 Affective responses

**Figure 1:**
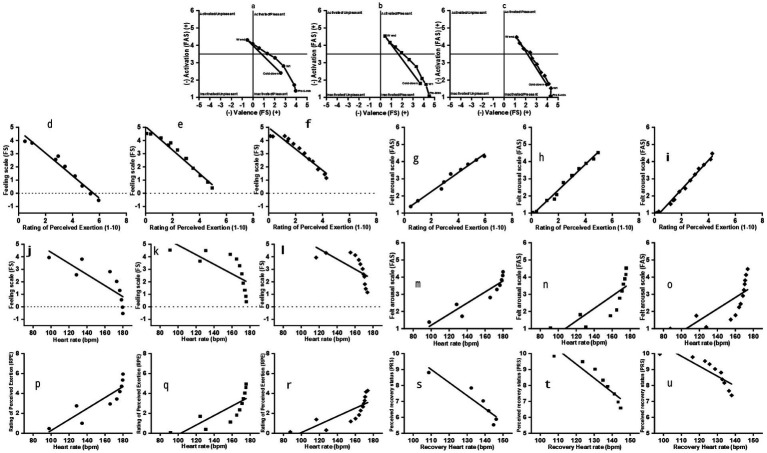
Correlations between perceptual during Lv-HIIE. Each point represents the mean affective or preceptual response across all participants at a work interval within the sessions. Session 1 (S1; ●), Session 15 (S15; ■), and Session 30 (S30; ♦).

Affective responses (valence and activation) during the work intervals for three sessions of Lv-HIIE protocols were plotted onto a circumplex model (Figures 1a–c). There was a shift from the inactivated/pleasant to the activated/pleasant quadrant for the Session 15 Lv-HIIE and Session 30 Lv-HIIE work intervals. During Session 1, Lv-HIIE, the affective responses shifted from inactivated/pleasant to the activated/unpleasant quadrant for the work intervals.

#### 3.1.2 Affective responses and perceived exertion

Figures 1d–f presents the correlations between FS and RPE across the three Lv-HIIE sessions. Strong negative correlations were identified in all sessions (Session 1: *r* = −0.98, 95% CI [−0.998, −0.823], *p* < 0.001; Session 15: *r* = −0.98, 95% CI [−0.997, −0.890], *p* < 0.001; Session 30: *r* = −0.97, 95% CI [−0.993, −0.874], *p* < 0.001). Across all sessions, FAS showed strong positive correlations with RPE (Session 1: *r* = 0.99, 95% CI [0.908, 0.999], *p* < 0.001; Session 15: *r* = 0.99, 95% CI [0.890, 0.997], *p* < 0.001; Session 30: *r* = 0.99, 95% CI [0.874, 0.993], *p* < 0.001; Figures 1g–i).

#### 3.1.3 HR and affective responses

Strong negative correlations (Session 1: *r* = −0.82, 95% CI [−0.977, −0.116], *p* = 0.007; Session 15: *r* = −0.71, 95% CI [−0.934, −0.063], *p* < 0.02; Session 30: *r* = −0.66, 95% CI [−0.912, −0.027], *p* < 0.02) were found between FS and HR across sessions (Figures 1j–l). However, inspection of the HR–FS scatterplots indicated that this association was not strictly linear and was better characterized by a negative quadratic function. Accordingly, quadratic regression analyses were additionally conducted to describe the non-linear pattern, with *R*^2^ reported to reflect model explanatory power rather than effect magnitude. Quadratic regression analyses yielded the following fitted equations: Session 1: FS = −8.26 × 10^−4^·HR^2^ + 0.192·HR − 7.29 (*R*^2^ = 0.77); Session 15: FS = −1.04 × 10^−3^·HR^2^ + 0.247·HR − 9.84 (*R*^2^ = 0.69); Session 30: FS = −9.65 × 10^−4^·HR^2^ + 0.232·HR − 9.04 (*R*^2^ = 0.66). Across all sessions, the consistently negative second-order coefficients indicate a downward-curving relationship, whereby affective valence declined more rapidly with increasing HR at lower-to-moderate intensities and tended to plateau at higher heart rate levels.

In contrast, FAS and HR remained strongly and positively correlated throughout the intervention (Session 1: *r* = 0.92, 95% CI [0.434, 0.993], *p* < 0.001; Session 15: *r* = 0.81, 95% CI [0.191, 0.960], *p* = 0.002; Session 30: *r* = 0.76, 95% CI [0.074, 0.947], *p* = 0.003; Figures 1m–o). Further analysis indicated that the HR–FAS relationship was better characterized by a positive quadratic function. The fitted equations were: Session 1, FAS = 3.71 × 10^−4^·HR^2^–0.0738·HR + 5.19 (*R*^2^ = 0.90); Session 15, FAS = 8.08 × 10^−4^·HR^2^–0.186·HR + 11.54 (*R*^2^ = 0.82); and Session 30, FAS = 8.82 × 10^−4^·HR^2^–0.204·HR + 12.56 (*R*^2^ = 0.76).

#### 3.1.4 HR and perceived exertion

Across the three Lv-HIIE sessions, HR was strongly and positively correlated with RPE (Session 1: *r* = 0.88, 95% CI [0.204, 0.984], *p* = 0.002; Session 15: *r* = 0.80, 95% CI [0.154, 0.956], *p* = 0.003; Session 30: *r* = 0.80, 95% CI [0.154, 0.946], *p* = 0.001; Figures 1p–r). Quadratic regression analyses further indicated that the HR–RPE relationship was non-linear and better described by a quadratic function, with the fitted equations as follows: Session 1, RPE = 6.15 × 10^−4^·HR^2^–0.121·HR + 6.73 (*R*^2^ = 0.82); Session 15, RPE = 9.31 × 10^−4^·HR^2^–0.208·HR + 11.74 (*R*^2^ = 0.77); and Session 30, RPE = 9.13 × 10^−4^·HR^2^–0.205·HR + 11.58 (*R*^2^ = 0.78).

#### 3.1.5 HRR and perceived recovery

Figures 1s–u shows the correlations between HRR and PRS across sessions. Strong negative correlations were observed in all sessions (Session 1: *r* = −0.93, 95% CI [−0.994, −0.468], *p* = 0.008; Session 15: *r* = −0.92, 95% CI [−0.989, −0.441], *p* = 0.001; Session 30: *r* = −0.84, 95% CI [−0.964, −0.214], *p* = 0.002).

### 3.2 Correlations between perceptual responses and cognitive–personality traits

Before the Lv-HIIE intervention, participants demonstrated moderate-to-high levels of goal orientation and personality hardiness, as reflected by mean GOEM and CD-RISC scores. Goal orientation was examined using the overall GOEM composite score; separate analyses of task and ego orientations were not conducted due to limited statistical power associated with the modest sample size. The mean total score for goal orientation (GOEM) was 31.28 ± 5.70, while the mean score for hardiness (CD-RISC) was 73.72 ± 9.80, indicating generally favorable cognitive–personality profiles prior to the intervention.

#### 3.2.1 Correlations between goal orientation and perceptual responses

**Figure 2:**
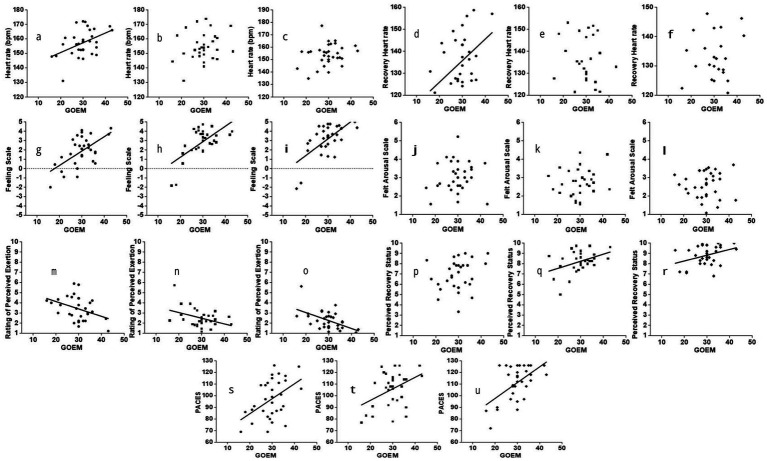
Correlations between goal orientations and perceptual responses across the three Lv-HIIE sessions. Session 1 (S1; ●), Session 15 (S15; ■), and Session 30 (S30; ♦).

Figures 2a–c presents the correlations between GOEM scores and HR across the three Lv-HIIE sessions. A moderate positive correlation was observed during the work intervals in Session 1 (*r* = 0.45, 95% CI [0.084, 0.723], *p* = 0.01), whereas no significant correlations appeared in Sessions 15 and 30 (*r* = 0.24 and 0.25, both *p* > 0.05). Across the three sessions, GOEM scores were moderately and positively associated with HRR, especially during recovery intervals in Session 1 (*r* = 0.43, 95% CI [0.059, 0.712], *p* < 0.02). No significant relationships were identified in Sessions 15 and 30 (*r* = 0.12 and 0.15, both *p* > 0.05; Figures 2d–f).

Strong and consistent associations were observed between GOEM scores and FS across sessions (Figures 2g–i). A moderate positive correlation was detected in Session 1 (*r* = 0.59, 95% CI [0.164, 0.846], *p* < 0.001), which increased to strong levels in Session 15 (*r* = 0.65, 95% CI [0.171, 0.891], *p* < 0.001) and Session 30 (*r* = 0.65, 95% CI [0.150, 0.886], *p* < 0.001). No significant relationships were recorded between GOEM scores and FAS during work intervals in Sessions 1, 15, and 30 (*r* = −0.01 to 0.14, all *p* > 0.05; Figures 2j–l).

Figures 2m–o illustrates the associations between GOEM scores and RPE across sessions. Weak negative correlations were observed in Session 1 (*r* = −0.39, 95% CI [−0.760, 0.248], *p* < 0.03) and Session 15 (*r* = −0.38, 95% CI [−0.745, 0.239], *p* = 0.03), while a moderate negative correlation emerged in Session 30 (*r* = −0.51, 95% CI [−0.832, 0.079], *p* = 0.003). In relation to PRS, no significant correlation was found in Session 1 (*r* = 0.25, 95% CI [−0.410, 0.752], *p* > 0.05). A moderate positive correlation appeared in Session 15 (*r* = 0.41, 95% CI [−0.304, 0.833], *p* = 0.02), and a weak positive correlation was noted in Session 30 (*r* = 0.36, 95% CI [−0.360, 0.812], *p* < 0.05; Figures 2p–r).

Correlations between GOEM scores and PACES during the three Lv-HIIE sessions are shown in Figures 2s–u. Moderate positive correlations were observed during the post-exercise period in all sessions (Session 1: *r* = 0.47, 95% CI [−0.231, 0.865], *p* = 0.007; Session 15: *r* = 0.40, 95% CI [−0.304, 0.833], *p* < 0.03; Session 30: *r* = 0.56, 95% CI [−0.075, 0.884], *p* < 0.001).

#### 3.2.2 Correlations between personality hardiness and perceptual responses

**Figure 3:**
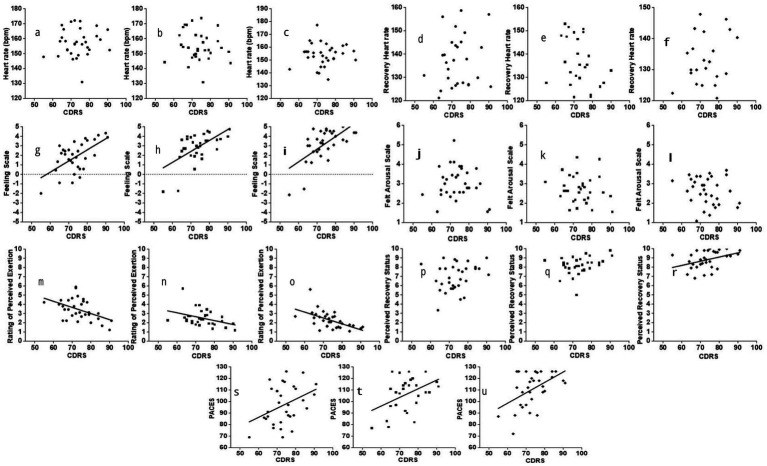
Correlations between personality hardiness and perceptual across the three Lv-HIIE sessions. Session 1 (S1; ●), session 15 (S15; ■), and session 30 (S30; ♦).

Across the three Lv-HIIE sessions, no significant correlations were observed between CDRS scores and HR during the work intervals (all *p* > 0.05, *r* = −0.11 to 0.11; Figures 3a–c). Similarly, CDRS scores showed no significant associations with HRR across all sessions (all *p* > 0.05, *r* = −0.22 to 0.11; Figures 3d–f).

Figures 3g–i displays the relationships between CDRS scores and FS. A moderate positive correlation was recorded in Session 1 (*r* = 0.59, 95% CI [−0.23, 0.92], *p* < 0.001), with strong positive correlations observed in Session 15 (*r* = 0.60, 95% CI [−0.03, 0.90], *p* < 0.001) and Session 30 (*r* = 0.64, 95% CI [0.02, 0.90], *p* < 0.001). No significant correlations were detected between CDRS scores and FAS across all sessions (*r* = −0.21 to −0.10, all *p* > 0.05; Figures 3j–l).

Moderate negative correlations were found between CDRS scores and RPE during the work intervals in Session 1 (*r* = −0.47, 95% CI [−0.884, 0.315], *p* = 0.007) and Session 30 (*r* = −0.56, 95% CI [−0.904, 0.179], *p* < 0.001), whereas a weak negative correlation appeared in Session 15 (*r* = −0.38, 95% CI [−0.863, 0.406], *p* < 0.001; Figures 3m–o). For PRS, no significant correlations were noted in Sessions 1 and 15 (*r* = 0.26 and 0.32, both *p* > 0.05). A weak positive correlation was observed in Session 30 (*r* = 0.39, 95% CI [−0.382, 0.811], *p* < 0.03; Figures 3p–r).

Correlations between CDRS scores and PACES during the three Lv-HIIE sessions are shown in Figures 3s–u. Weak positive correlations were observed during the post-exercise period in Session 1 (*r* = 0.38, 95% CI [−0.406, 0.812], *p* < 0.04) and Session 15 (*r* = 0.36, 95% CI [−0.420, 0.806], *p* = 0.04). A moderate positive correlation was observed in Session 30 (*r* = 0.49, 95% CI [−0.299, 0.858], *p* = 0.004).

## 4 Discussion

The key findings from this study are as follows: (1) Significant correlations were observed among perceptual responses during Lv-HIIE, with affective valence negatively associated with heart rate and perceived exertion, and positively associated with enjoyment across sessions; (2) Cognitive–personality traits, including goal orientation and hardiness, showed moderate to strong positive correlations with affective valence and enjoyment, and negative correlations with perceived exertion, indicating that individuals with higher levels of these traits reported more positive affective experiences and greater enjoyment during Lv-HIIE; (3) No significant associations were found between cognitive–personality traits and physiological indicators (heart rate or heart rate recovery), suggesting that these traits primarily relate to perceptual rather than physiological responses; (4) Overall, these findings indicate that cognitive–personality traits are meaningfully associated with perceptual regulation and affective adaptation during Lv-HIIE, extending previous evidence that motivational and resilience-related factors modulate affective responses during high-intensity exercise.

The present findings indicate a coherent pattern of associations among perceptual and physiological indicators during Lv-HIIE, reflecting dynamic interactions between affective, exertional, and physiological responses across training progression. The inverse relationships between affective valence and both perceived exertion and heart rate suggest that greater physiological strain and perceived effort tend to coincide with less positive affect, which is consistent with the Dual-Mode Theory (Ekkekakis et al., 2005). This pattern likely reflects the influence of interoceptive cues, such as breathlessness and muscle fatigue, particularly when exercise intensity exceeds the ventilatory threshold.

In contrast, the positive associations observed among felt arousal, heart rate, and perceived exertion indicate that physiological activation and subjective effort increase concurrently, aligning with previous evidence linking affective arousal to cardiovascular activation (Schwarz et al., 2013; Cellini et al., 2023). Notably, the strength of these associations appeared to attenuate over the 10-week Lv-HIIE intervention, potentially reflecting both physiological and psychological adaptation. As participants became more familiar with the exercise protocol, improved tolerance to physical strain and enhanced emotional regulation may have contributed to the reduced coupling between heart rate and affective valence (*r* = −0.82 to −0.66), heart rate and perceived exertion (*r* = 0.88 to 0.80), and heart rate recovery and perceived recovery status (*r* = −0.93 to −0.84). These trends are broadly consistent with previous findings indicating that affective responses become more positive with increased fitness and repeated exposure to high-intensity exercise (Malik et al., 2018; Oliveira et al., 2018). Moreover, the observed patterns may also reflect improvements in cardiovascular recovery and autonomic regulation over time (Molina et al., 2016; Barrero et al., 2020; Di Credico et al., 2024).

Taken together, these results indicate a coordinated pattern across affective, exertional, and physiological domains, reflecting a psychological affective–exertional coupling, defined as the co-variation of emotional experiences with perceived exertional and physiological responses during Lv-HIIE (Williams et al., 2012; Ekkekakis et al., 2020; Farias-Junior et al., 2020). Moreover, repeated exercise exposure was associated with changes in this coupling, potentially reflecting adaptive emotional regulation over time.

Despite these coherent patterns, several important limitations should be acknowledged. First, the modest sample size (*n* = 32) may limit the generalizability of the present findings to broader populations of adults with overweight and obesity. Although determined *a priori* and sufficient to detect moderate associations, the sample size may not fully capture inter-individual variability in perceptual and physiological responses to Lv-HIIE. Moreover, while the sample size was adequate for the correlational analyses conducted in the present study, it may not be sufficient for establishing robust cause-and-effect relationships or for applying more sophisticated analytical approaches, such as structural equation modeling. Second, the sample exhibited an unbalanced sex distribution, with a greater proportion of females than males. Although sex-specific analyses were not conducted due to limited statistical power, existing evidence regarding sex differences in affective and perceptual responses to high-intensity interval exercise is mixed. Specifically, some studies report no sex-related differences when physiological capacity is matched, whereas others indicate more positive affective responses in females under certain conditions (Coe and Astorino, 2024; Astorino and Sheard, 2019). Accordingly, potential sex-related influences on perceptual responses during Lv-HIIE cannot be ruled out and warrant further investigation in larger, sex-balanced samples. Third, the correlational nature of the analyses precludes causal inference regarding the relationships among cognitive–personality traits, physiological indicators, and perceptual responses; thus, the observed associations should be interpreted as descriptive rather than mechanistic. In addition, this design does not permit more advanced analytical approaches, such as structural equation modeling, to examine indirect or mediating pathways. Fourth, repeated measurements across multiple training sessions were analyzed without explicitly accounting for within-subject dependency, which may have led to underestimated standard errors and inflated association estimates. Future studies employing multilevel or mixed-effects models may provide a more rigorous framework for capturing both within- and between-subject variability over time.

The present findings further indicate that cognitive–personality traits, namely goal orientation and hardiness, are meaningfully related to perceptual experiences during Lv-HIIE. Specifically, both traits showed consistent positive associations with affective valence and post-exercise enjoyment, and negative associations with perceived exertion. For example, GOEM and CDRS were positively correlated with FS (*r* = 0.59–0.65, *p* < 0.001) and PACES (*r* = 0.38–0.56, *p* < 0.05), while both were negatively correlated with RPE (*r* = −0.38 to −0.56, *p* < 0.01). These findings suggest that individuals with stronger goal orientation and greater psychological hardiness experienced higher in-task pleasure and enjoyment, alongside lower perceived exertion. This pattern is consistent with previous research indicating that goal orientation is positively associated with exercise enjoyment and engagement (Easton et al., 2021), and that achievement goal orientations are linked to emotional outcomes in physical activity (Biddle et al., 2003; Ntoumanis and Biddle, 1999; Stuntz and Weiss, 2009). Extending this literature, the present study demonstrates that these associations persisted across multiple Lv-HIIE sessions, suggesting a stable cognitive–affective coupling with repeated exposure that may contribute to improved affective regulation and exercise adherence over time.

These patterns may be explained by cognitive appraisal and coping processes that shape how individuals interpret exercise-induced strain. Task-oriented individuals typically report greater happiness and wellbeing (Briki, 2020; Martínez-González et al., 2021), while personality characteristics such as conscientiousness and resilience may influence the relationship between personality and goal orientation (Tomczak et al., 2024). In this context, exercise-related strain may be appraised as a challenge rather than a threat, thereby reducing perceived exertion and emotional stress (Eschleman et al., 2010). However, goal orientation and self-efficacy alone may not fully account for perceived exertion, as other psychological factors, including coping strategies and perceived competence, are also likely to contribute (Tenebaum et al., 2001). Overall, the present findings suggest that cognitive–personality traits partially explain individual variability in affective and exertional responses during Lv-HIIE.

The present findings highlight both theoretical and practical implications by linking cognitive–personality traits to perceptual regulation during Lv-HIIE. Extending the Dual-Mode Theory (Ekkekakis et al., 2005), the results indicate that goal orientation and hardiness are associated with affective and exertional responses, as participants with higher GOEM and CDRS scores reported more positive affective valence and enjoyment (*r* = 0.59–0.65; *r* = 0.38–0.56) alongside lower perceived exertion (*r* = −0.38 to −0.56). These findings suggest that individuals with stronger goal orientation and greater resilience may reinterpret physiological strain as a challenge, thereby sustaining more positive affective states under high-intensity stress.

From a practical perspective, psychological profiling may support more individualized Lv-HIIE prescription and promote exercise adherence among adults with overweight and obesity. Although the present study employed a multi-session Lv-HIIE intervention, future research using designs that allow stronger causal inference (e.g., randomized comparisons or mediation-based frameworks), in combination with neurophysiological and hormonal markers, may further elucidate the mechanisms underlying perceptual adaptation. In addition, studies with larger samples may benefit from examining task- and ego-oriented goal orientations separately to clarify their distinct roles in perceptual regulation during high-intensity exercise. Collectively, these findings contribute empirical support for the role of cognitive–personality traits in shaping perceptual regulation during exercise and underscore their relevance for the development of psychologically informed and sustainable Lv-HIIE interventions.

## 5 Conclusion

In conclusion, our findings comprehensively extend previous work on perceptual and psychological regulation during HIIE and indicate that cognitive–personality traits, particularly goal orientation and hardiness, are key modulators of perceptual responses during Lv-HIIE in overweight-to-obese adults. Individuals with higher goal orientation and greater hardiness consistently reported more positive affective valence and enjoyment, together with lower perceived exertion, suggesting a stable cognitive–affective coupling that facilitates adaptive regulation under physiological strain. Furthermore, moderate-to-strong intercorrelations among perceptual indicators (e.g., affective valence, arousal, RPE, recovery, and enjoyment) highlight a coordinated psychophysiological network that evolves dynamically with repeated exercise exposure. These findings collectively suggest that resilient and goal orientation individuals are more capable of reinterpreting exertional stress as a manageable challenge, sustaining positive affect and engagement across sessions. Although mechanistic data on neurophysiological and hormonal pathways were not included in this analysis, the observed patterns provide preliminary insight into how cognitive–affective regulation contributes to exercise tolerance and enjoyment. Therefore, our findings support the incorporation of psychological profiling and cognitive–affective training components in Lv-HIIE prescription to enhance adherence, tolerance, and overall exercise experience in overweight-to-obese adults.

## Data Availability

The raw data supporting the conclusions of this article will be made available by the authors without undue reservation.
